# Correction: Inhibition of PAD4 enhances radiosensitivity and inhibits aggressive phenotypes of nasopharyngeal carcinoma cells

**DOI:** 10.1186/s11658-023-00444-x

**Published:** 2023-04-04

**Authors:** Hao Chen, Min Luo, Xiangping Wang, Ting Liang, Chaoyuan Huang, Changjie Huang, Lining Wei

**Affiliations:** 1 Department of Oncology, The Second Nanning People’s Hospital, No. 13 Dancun Road, Jiangnan District, Nanning, 530031 Guangxi China; 2 Department of Endoscopy, The Affiliated Tumor Hospital of Guangxi Medical University, Nanning, 530021 Guangxi China

**Correction: Cellular & Molecular Biology Letters 2021, 26(1):9 ** 10.1186/s11658-021-00251-2

Following publication of the original article [1], the authors informed us that the images of Transwell assays (Figs. 2G, 3F, 4F) were incorrect. The correct images are given below. The replacement of images does not affect the original conclusion.

**Fig. 2:**
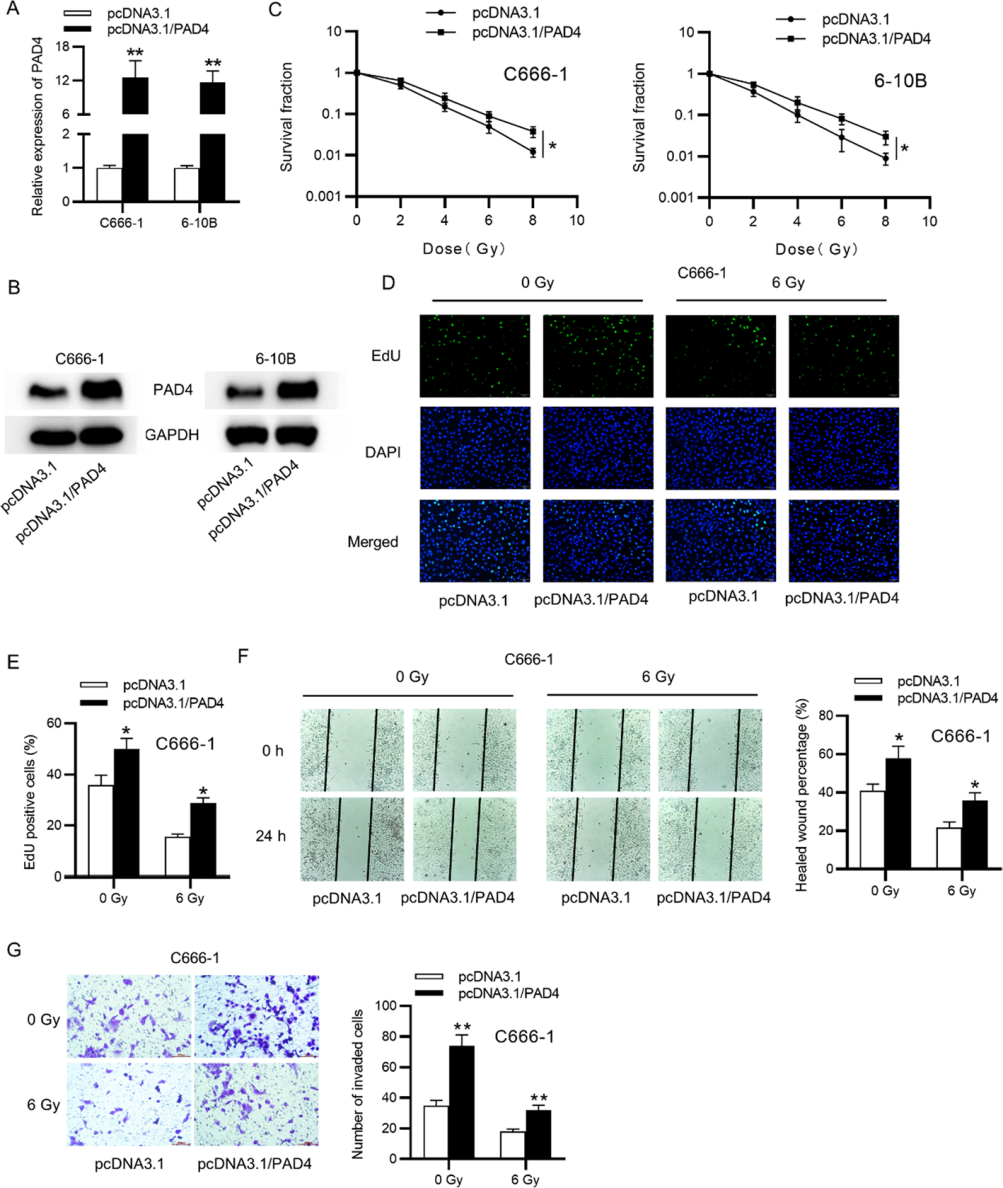
PAD4 overexpression promotes the radioresistance and cellular processes in NPC. **A**, **B** The PAD4 level in NPC cells transfected with pcDNA3.1/PAD4 by RT-qPCR and western blot. **C** Colony formation assay for cell survival under diferent doses of radiation. **D**, **E** Cell proliferation in NPC cells transfected pcDNA3.1/PAD4 by EdU assay, at 24 h after 6 Gy irradiation. **F**, **G** Migration and invasion in NPC cells transfected pcDNA3.1/PAD4 by wound healing and Transwell, at 24 h after 6 Gy irradiation. Unpaired Student’s test. *p < 0.05, **p < 0.01

**Fig. 3:**
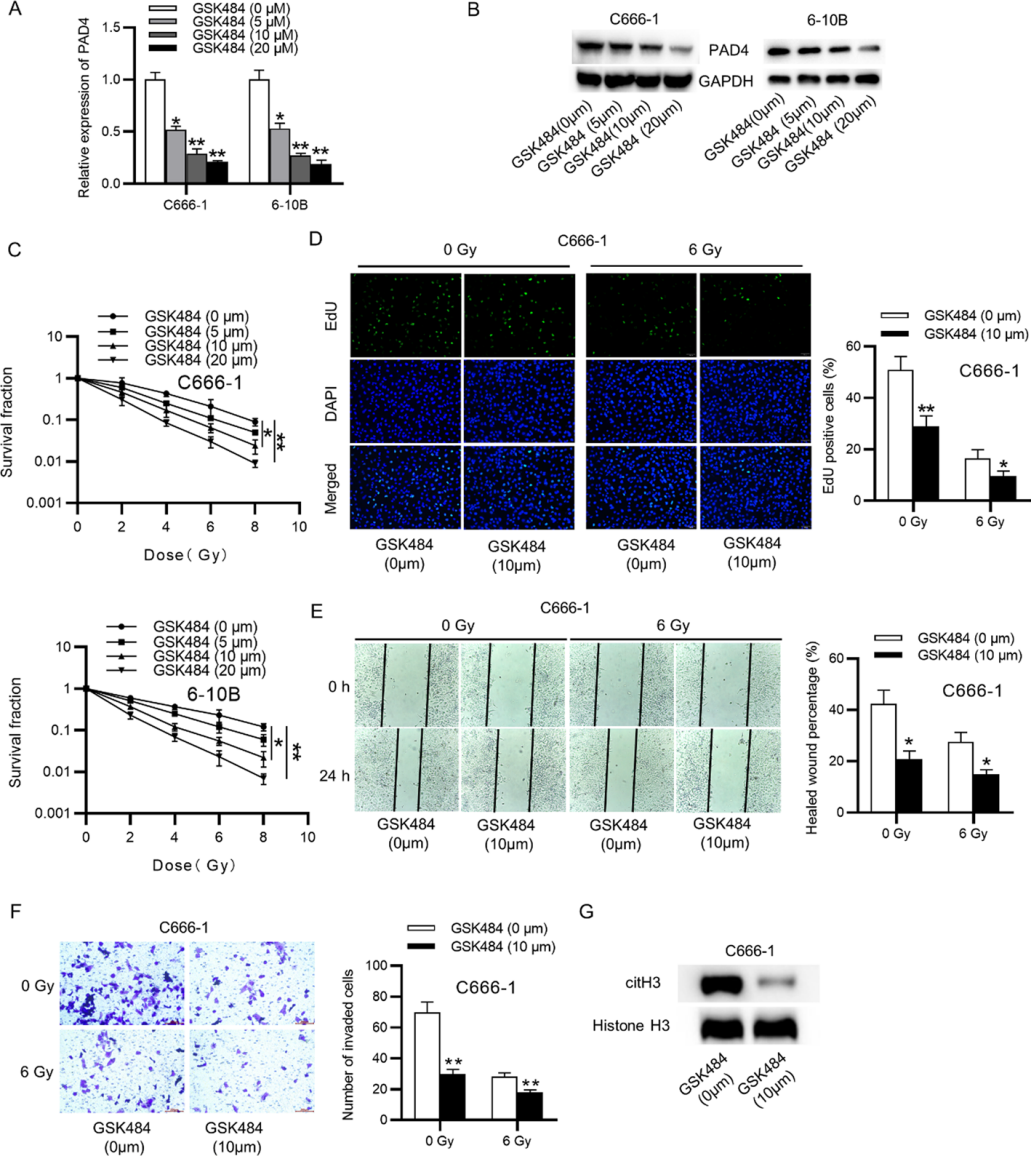
Inhibition of PAD4 promotes the radiosensitivity and cellular processes in NPC. **A**, **B** The PAD4 level after treatment with GSK484 by RT-qPCR and western blot. **C** Colony formation assay of NPC cells treated with diferent concentrations of GSK484. One-way ANOVA with Tukey’s post hoc test. **D** Cell proliferation in NPC cells treated with GSK484 or not by EdU assay, at 24 h after 6 Gy irradiation. **E**, **F** Migration and invasion in NPC cells treated with GSK484 or not by wound healing and Transwell assays, at 24 h after 6 Gy irradiation. **G** The level of citH3 protein in NPC cell treated with GSK484 or not was measured by western lot. Unpaired Student’s test. *p < 0.05, **p < 0.01

**Fig. 4:**
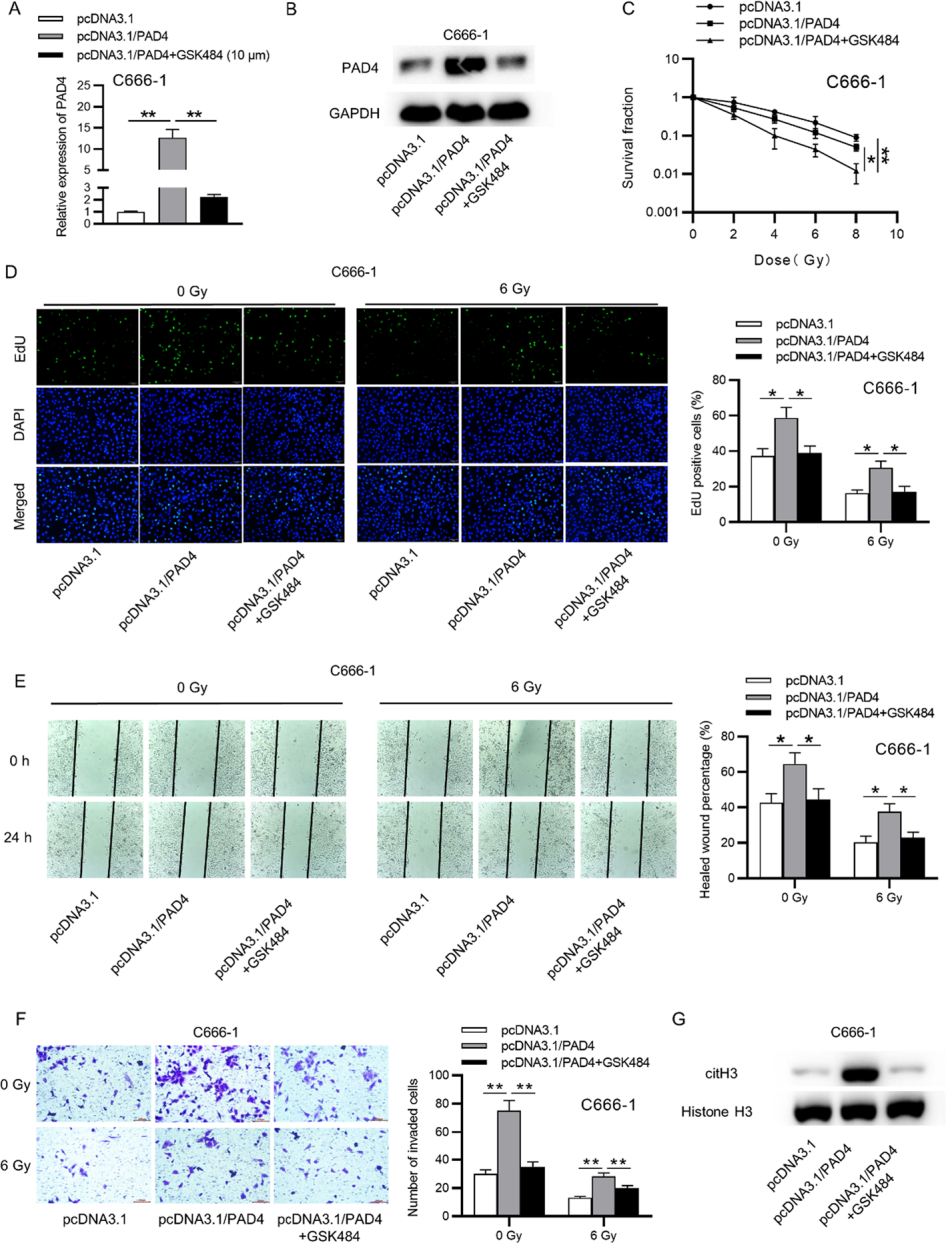
Treatment of GSK484 attenuates the efect of PAD4 overexpression of NPC cell functions. **A**, **B** The PAD4 level in NPC cells treated diferently by RT-qPCR and western blot analyses. **C** Colony formation assay for the survival of NPC cells treated diferently. **D** Cell proliferation in NPC cells treated diferently by EdU assay. **E**, **F** Migration and invasion ability in NPC cells treated diferently by wound healing and Transwell. **G** The citH3 protein levels in NPC cell treated diferently by western lot. One-way ANOVA with Tukey’s post hoc test. *p < 0.01
